# Characteristics of the litter dynamics in a Moso bamboo forest after strip clearcutting

**DOI:** 10.3389/fpls.2022.1064529

**Published:** 2022-12-08

**Authors:** Yaxiong Zheng, Shaohui Fan, Fengying Guan, Xuan Zhang, Xiao Zhou

**Affiliations:** ^1^ Key Laboratory of National Forestry and Grassland Administration, International Center for Bamboo and Rattan, Beijing, China; ^2^ National Location Observation and Research Station of the Bamboo Forest Ecosystem in Yixing, National Forestry and Grassland Administration, Yixing, China

**Keywords:** Moso bamboo forest, strip clearcutting, litter biomass, nutrient content, litter decomposition

## Abstract

**Introduction:**

The quality of new Moso bamboo trees has been found to decrease in the years following strip cutting (SC) events. It is thus essential that we improve our knowledge of nutrient return after strip cutting in Moso bamboo forests to help facilitate sustainable management.

**Methods:**

In this investigation the dynamics of nutrient return were monitored in plots with 8 m wide strip cutting (SC), their reserve belts (RB), and a traditionally managed forest (CK) as the control, for 5 years after cutting.

**Results:**

The results showed that strip cutting significantly reduced nutrient return (*p<* 0.05), but as the plots recovered, the nutrient levels also recovered to match those of the control. The high densities in the RB no longer increase nutrient return. Five years after SC there was no significant difference in nitrogen and phosphorus returns among the three treatment plots, but potassium returns in the SC plot were significantly higher than those in the RB (*p<* 0.05). From 2–5 years after cutting, the litter decomposition rate in the RB was significantly higher than in the SC and CK (*p<* 0.05). In addition, the decomposition rate in the SC plot was significantly accelerated five years after logging, which suggests that long-term strip cutting management may lead to the restriction of nutrients on the growth and development of new trees.

**Discussion:**

The results indicate that nutrients should be added *via* artificial fertilization in the future.

## Introduction

Moso bamboo (*Phyllostachys edulis*) is one of the most important forest resources in China as it has both economic and ecological value (Jiang, 2007). However, due to the development of the social economy and urbanization of the population there is a labor shortage which has increased cutting costs, and this has subsequently reduced the enthusiasm of farmers to engage with this industry (Fan et al., 2018). Experts and scholars have proposed a strip clearcutting model as a means by which to reduce logging costs (Fan et al., 2018; Zeng, 2019). Studies on the restoration dynamic characteristics of Moso bamboo under different cutting widths have shown that cutting increased the number of shoots per unit area and decreased the quality of new bamboo trees, and the results unanimously found that 6–9 m was the optimal cutting width (Zeng, 2019). Strip cutting removes a large amount of nutrients from the forest at one time, and this disturbs the nutrient cycling and overall balance of the bamboo forest ecosystem (Zheng et al., 2022c). The subsequent reductions in new tree quality may reflect the overall degradation in bamboo forest productivity after cutting (Liu, 2009). Our previous study has found that although there was no significant difference in stand density between SC and CK after 5 years, the average bamboo height and average diameter at breast height (DBH) in SC were lower than those in CK (Zheng et al., 2022b). The development of the bamboo forests mainly depends on the nutrient cycling of soil organic matter to maintain fertility (Zheng et al., 2022c). Strip logging may thus increase concerns about long-term productivity loss (Turner and Lambert, 2013).

The cycling and balance of nutrients in an ecosystem affects its productivity, stability, and sustainability (Johnson and Turner, 2019). Furthermore, the exchange of matter and energy through interactions with the environment is fundamental to the survival and development of all living things (Tu et al., 2014). Further research on nutrient cycling could thus help to clarify the mechanisms of material cycling in specific ecosystems and guide production practices to help improve ecosystem limiting factors and productivity (Vitousek, 1984). Litter is the main linking factor between vegetation and the soil mineral content, and it thus plays a central role in nutrient cycling in forest ecosystems (Huang et al., 2017). By assessing the differences in litter yield, we can understand the impacts of logging on the ecological functions of the Moso bamboo community (Zhou et al., 2007). Data on the biomass, nutrient content, decomposition rate, and other factors are required to study nutrient cycling (Huang et al., 2017; Zheng et al., 2021b). The nutrient content of the litter directly affects the quality and decomposition rate of nutrients and this indirectly affects plant root uptake (Ren et al., 2018). The return of nutrients to the soil through litter decomposition is an important ecological process (Zheng et al., 2022c). It has been reported that litter provides more than 70% of plant growth nutrients through nutrient deposition (Adolfo et al., 2016). Many factors affect the decomposition rate of litter, including temperature (Bothwell et al., 2014), precipitation (Wieder et al., 2009), litter matrix quality (Astel et al., 2009), and soil nutrient availability (Keeler et al., 2009). Most studies have shown that the biogeochemical processes after harvesting are altered due to changes in species composition, root uptake requirements, soil conditions, and microbial activities (Lilli et al., 2018).

The long-term nutrient return dynamics of bamboo forests after strip cutting are still unclear, but this knowledge is critical for the development of efficient nutrient management practices and harvesting patterns for Moso bamboo forests. In this investigation we have studied the litter yield and nutrient content dynamics in Moso bamboo forests after strip clearcutting to reveal the differences in litter decomposition rates and provide a theoretical basis for the nutrient and long-term management of bamboo. We hypothesized that: (1) strip cutting would reduce the density of bamboo forests and have a negative effect on nutrient return; (2) that high densities in reserved belts may no longer increase nutrient return; and (3) that cutting may affect the litter decomposition rate.

## Materials and methods

### Study site

**Figure 1:**
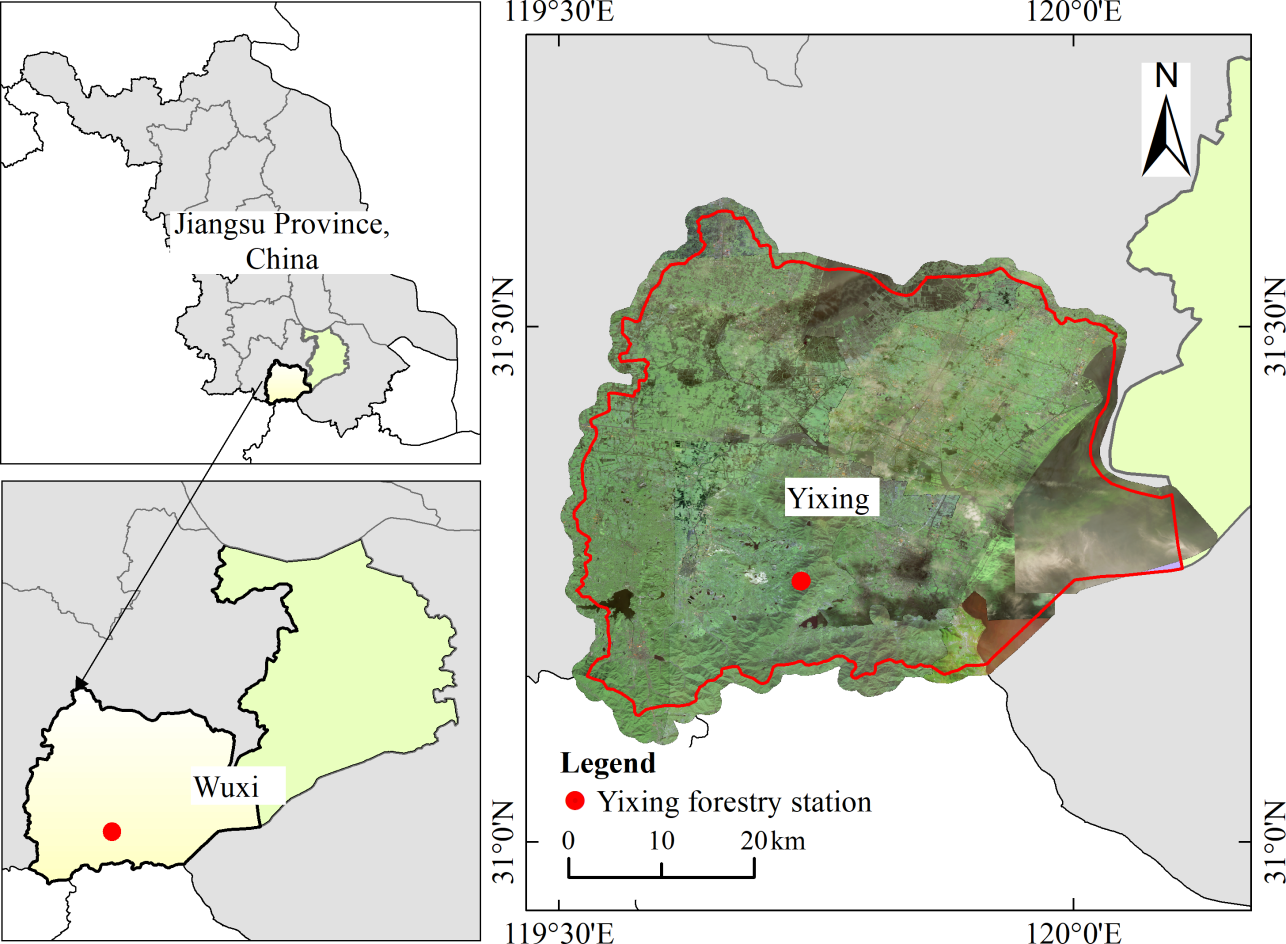
Location of the study area.

The study was conducted on the Yixing forest farm in southern Jiangsu Province, China (**Figure 1**) (Zheng et al., 2022b). The experimental area is within the marine monsoon climate zone in the subtropical region. The lowest temperature is -4.5°C and the highest is 38.8°C, and the average annual and monthly temperatures are 16.5°C and 28.3°C, respectively (Zheng et al., 2021b). Rainfall occurs throughout the four seasons, with an average annual precipitation of 1229.9 mm. The terrain is dominated by low hills. The predominant understory species include *Oxalis corniculata*, *Hedyotis chrysotricha*, *Paederia cruddasiana*, and *Salvia prionitis* (Zheng et al., 2022a).

### Experimental design

Strip clearcutting refers to cutting all bamboo in the plot. In February 2017, pure Moso bamboo stands with the same management measures, slope, and the same stand structure were selected for the experiment. Three strip cut plots (SC) with a width of 8 m and a length of 20 m were established. Reserved plots (RB) with the same width were set on both sides of the SC. The function of the reserved plots was to transport nutrients for the growth and development of the new bamboo in the SC through the underground whip root system. Meanwhile, isolation trenches 50-cm wide and 50-cm deep were dug around the plots to eliminate the effect of the physiological integration of Moso bamboo through long-distance nutrient transport (Zheng et al., 2021b). Three 20 × 20 m traditional management plots were set as the control (CK). The CK plots followed the original management methods including digging bamboo shoots and artificial selective harvest of old bamboo. The SC and RB were restored naturally, and no management measures were taken during the experiment. A continuous survey and sampling were conducted from the three treatment plots from 2017 to 2021. The basic information of the plot before cutting is shown in **Table 1**.

**Table 1:** Basic information of the plot before cutting.

Plot	Slope	Altitude	Density	Mean DBH	Mean treeheight	Soil total nitrogen	Soil total phosphorus	Soil total potassium
		(m)	(individual/hm^2^)	(cm)	(m)	(TN mg/g)	(TP mg/g)	(TK mg/g)
SC	5°	113	3432 ± 372a	8.77 ± 0.33a	13.29 ± 0.43a	1.41 ± 0.07a	0.26 ± 0.006a	9.32 ± 0.07a
RB	6°	113	3651 ± 184a	9.03 ± 0.30a	13.20 ± 0.34a	1.41 ± 0.19a	0.26 ± 0.000a	9.31 ± 0.08a
CK	5°	114	3787 ± 148a	8.91 ± 0.15a	13.39 ± 0.22a	1.40 ± 0.10a	0.26 ± 0.006a	9.34 ± 0.20a

Values represent the mean ± standard deviation (n = 3). Different lowercase letters indicate a significant difference in stand characteristics between the different treatment plots (p< 0.05).

### Leaf litter biomass survey and sampling

In March 2017, three litter traps that were each 1m × 1 m were placed in plots in the upper, middle, and lower levels. The litter traps were 1 m above the ground to ensure that the gauze net was large enough. At the same time, a stone was placed in the gauze net to avoid the wind blowing away the collection. From March 2017 to December 2021, litter was collected from each trap at the beginning of each month, dried at 65°C to a constant weight, and then weighed. After drying, the samples were ground in a fine powder, passed through a 2 mm (10 mesh) sieve, and stored in a sealed bag, and the relevant sampling information determined for nitrogen, phosphorus, and potassium content was marked on the bag.

### Decomposition of litter

In February 2017, the collected leaf litter was used to make leaf litter decomposition bags. The litter decomposition bags were 20 cm × 30 cm and made from 1 mm mesh nylon mesh. Twenty grams of dry leaf litter was initially placed in each bag. Then, 12 of the leaf litter decomposition bags were randomly placed in each plot, and there were a total of 108 leaf litter decomposition bags overall. During the placement, the litter on the surface was removed, and the bags were attached to the soil and fixed with PVC pipes. Three litter decomposition bags were collected from each plot every 3 months. The recycled litter bags were washed with clean water to remove the excess soil, dried at a constant temperature of 65°C, and then weighed. The decomposition rate was calculated using the negative exponential decay model proposed by Olson (Olson, 1963), with Equations 1-3. The setting of litter decomposition bags and sample treatment methods from 2018 to 2021 are consistent with those in 2017.


(1) $$\frac{M_{t}}{M_{o}}=ae^{-kt}$$

(2) $$t_{0.5}=-\ln\left(0.5\right)/k$$

(3) $$t_{0.95}=-\ln\left(0.05\right)/k$$

Where M_0_ is the initial dry mass of litter, M_t_ is the remaining dry mass of litter decomposed at time t (in years), k is the annual decay constant, t_0.5_ is the time of 50% mass loss, and t_0.95_ is the time of 95% mass loss.

### Chemical analysis

The mineral elements needed for the growth and development of Moso bamboo include N, K, P, Si, Ca, Zn, and B (Zheng et al., 2022c). In addition to nitrogen, phosphorus, and potassium, the contents of other elements in the soil can meet the growth of Moso bamboo (Su, 2012). Therefore, studies on the nutrients of Moso bamboo have mainly focused on nitrogen, phosphorus, and potassium. The total nitrogen (TN) content was determined using an elemental analyzer (ECS 4024 CHNSO; Costech, Picarro, Italy). Total phosphorus (TP) content was determined following the molybdenum-antimony resistance colorimetric method (concentrated H2SO4-HClO4) using an automatic chemical analyzer (Smartchem 300; AMS, Italy). Total potassium (TK) content was determined using a flame photometer (M410; Sherwood, United Kingdom).

### Statistical analysis

The differences in litter biomass, nutrient content, and nutrient return among different treatment plots were tested using One-way analysis of variance (ANOVA). The assumptions of normality and homogeneous variance were examined using the Shapiro-Wilk test and Leven’s test, respectively. The means were separated by the least significant difference (LSD) test, and statistical significance was set at *p*<0.05. All statistical analyses were performed in R (version 3.6.2), and the data were calculated using Excel 2016. All graphs were drawn using the ggplot2 package.

## Result

### Litter yield

**Figure 2:**
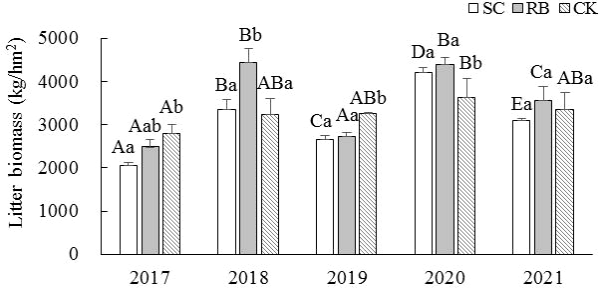
Dynamics of litter biomass in strip cut plot (SC), reserved belt (RB), and control plots (CK). Error bars represent standard deviation (n=3). Different capital letters indicate a significant difference in litter biomass between the different treatment plots at the same time (*p <* 0.05); Different lowercase letters indicate a significant difference in litter biomass between the different times in the same treatment plot (*p <* 0.05).

For the SC and RB plots, the litter yield during the off-year was significantly higher than that during the on-year (*p<* 0.05) (**Figure 2**). In 2017, the litter yield in the SC was significantly lower than that in the CK (*p<* 0.05). In 2018, the litter yield in the RB was significantly higher than that in the SC and CK (*p<* 0.05). In 2019, the litter yield in the CK was significantly higher than that in the SC and RB (*p<* 0.05). The litter yield in the CK in 2020 was significantly lower than that of SC and RB (*p<* 0.05). In 2021, there was no significant difference in litter yield among the three different treatment plots.

### Litter nutrient content

**Figure 3:**
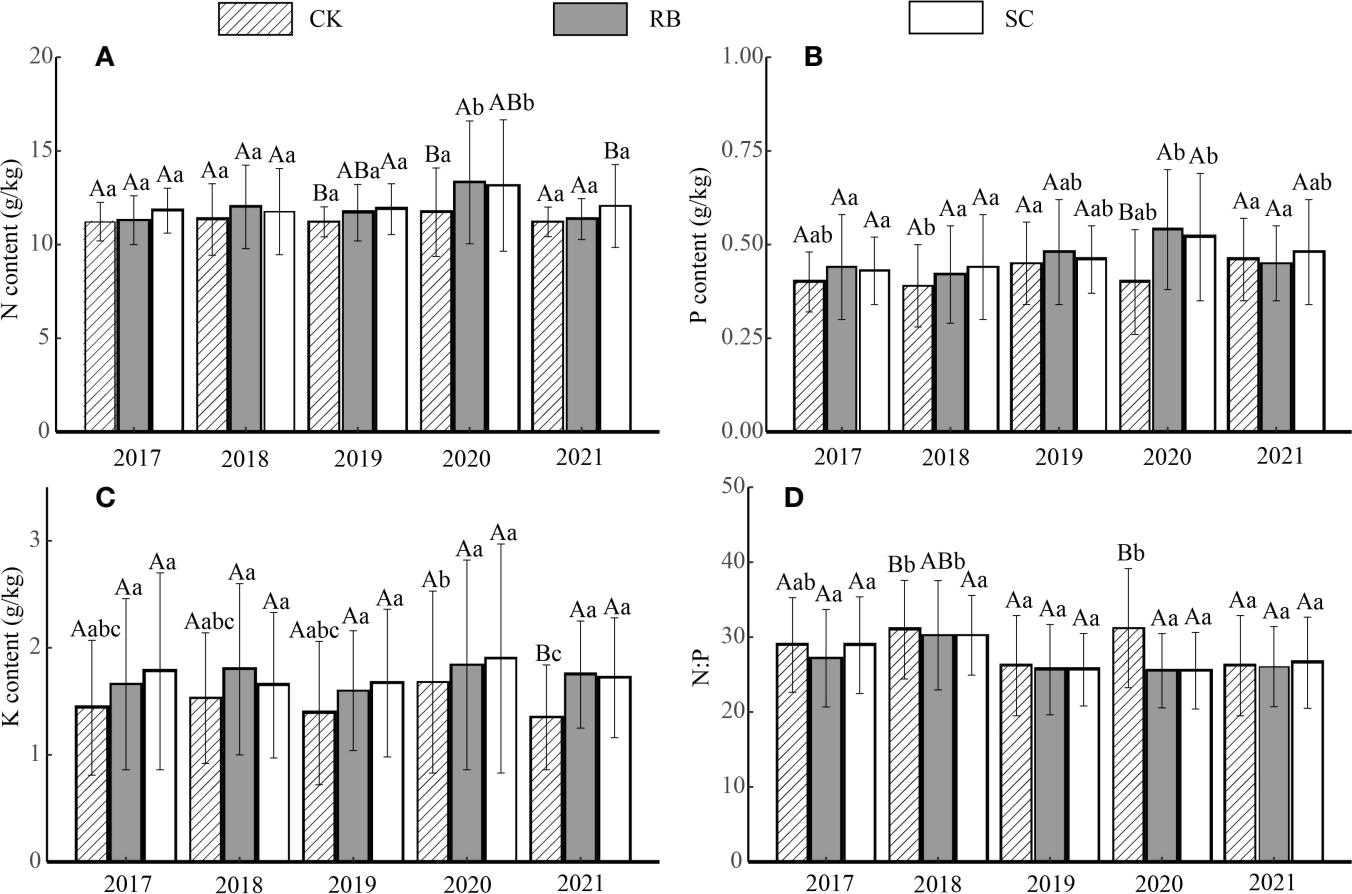
Nutrient content **(A)**, nitrogen; **(B)** phosphorus; **(C)** potassium; **(D)**, the ratio of nitrogen and phosphorus of litter in strip cut plot (SC), reserved belt (RB), and control plots (CK). Error bars represent standard deviation (n=3). Different capital letters indicate a significant difference in nutrient content between the different treatment plots at the same time (*p <* 0.05); Different lowercase letters indicate a significant difference in nutrient content between the different times in the same treatment plot (*p <* 0.05).

The nitrogen, phosphorus, and potassium content levels in the litter were not significantly affected at the early stage after cutting, but after 3 years, the nitrogen content in the litter was significantly higher than that in the CK (**Figure 3A**, *p<* 0.05). Four years after the cutting, the litter phosphorus content in the RB and SC increased significantly (**Figure 3B**, *p<* 0.05). Five years after the cutting, the N and K contents of the SC litter were significantly higher than that of the CK (**Figures 3A** and **C**, *p<* 0.05). There was no significant difference in litter nitrogen content over time among the three treatments. The potassium content in the CK litter in 2021 was significantly lower than that in 2020 (**Figure 3C**, *p<* 0.05). In the second and fourth years after cutting, the litter N:P value in the SC was significantly lower than that in the CK (**Figure 3D**, *p<* 0.05).

### Nutrient return

**Figure 4:**
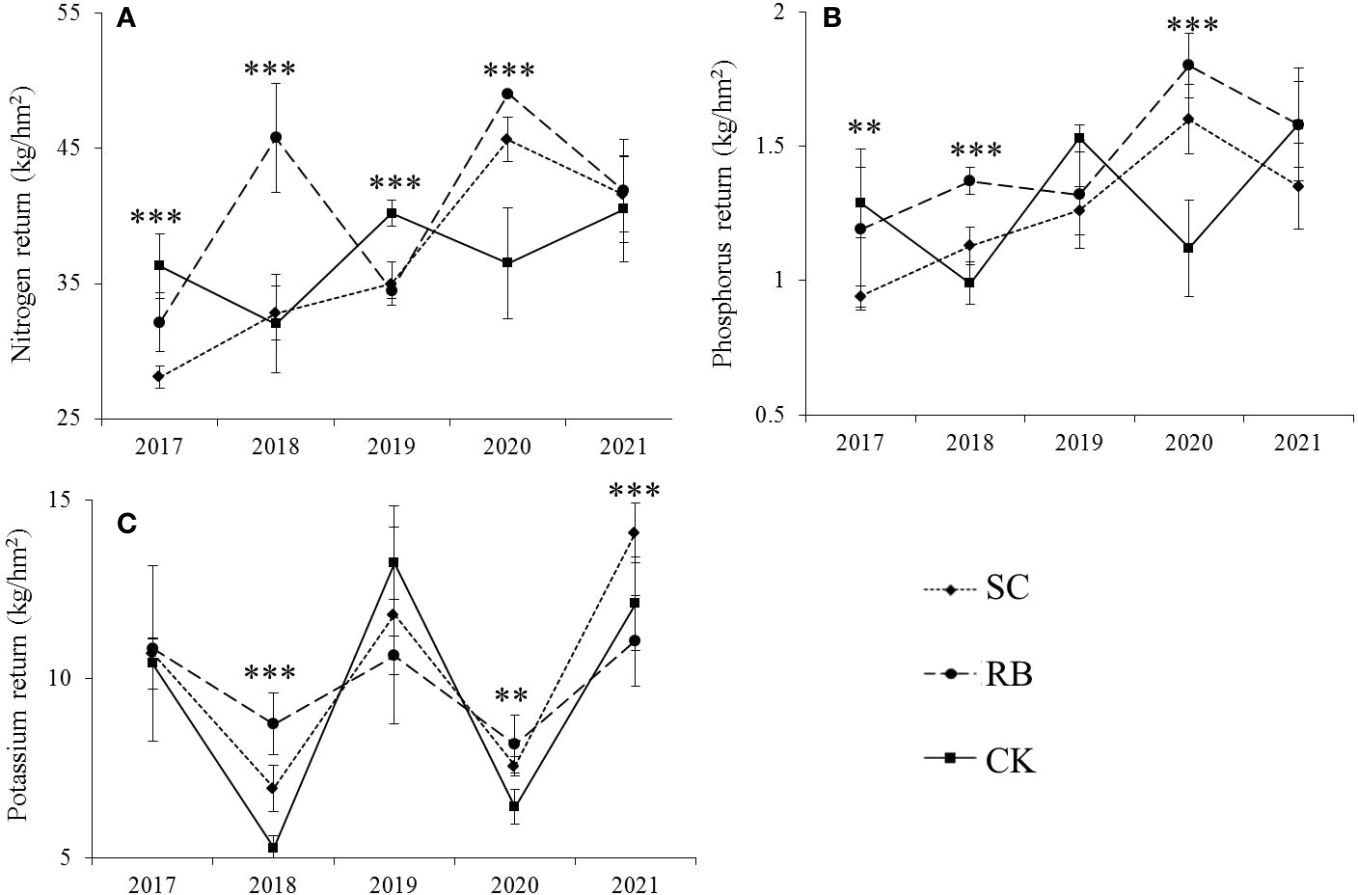
The nutrient return **(A)** nitrogen; **(B)** phosphorus; **(C)** potassium *via* litter to forest soil in strip cut plot (SC), reserved belt (RB), and control plots (CK). Error bars represent standard deviation (n=3). Asterisks indicate differences between study sites at different levels of significance: *p ≤* 0.001=***p ≤ 0.01=**p ≤ 0.05 =*.

Cutting significantly reduced the amount of N and P returned to the bamboo stands in the first year after cutting (*p<* 0.05), and the nitrogen and phosphorus returned showed CK > RB > SC (**Figure 4**). In the third year after cutting, the nutrient return in the CK was greater than that in the SC and RB. After five years, there was no significant difference in the nitrogen and phosphorus returns among the three treatment plots, but potassium returns in the SC were significantly higher than those in the RB (*p<* 0.05). In the off-year, the nutrient returns in the RB were greater than those in the SC and CK. As time passed, the amount of nitrogen and phosphorus in the SC gradually increased. The returns of the nitrogen and phosphorus in the RB increased in the off-year and decreased in the on-year, and in the CK they showed an opposite trend to that of the RB. The amount of K returned during the on-year was greater than that in the off-year.

### Litter decomposition

Strip cutting had no significant effect on the litter decomposition rate (**Table 2**). The litter decomposition rate in the SC and RB were the lowest in 2018 and highest in 2021. The decomposition rate of the litter in the CK did not change significantly with time. From 2018 to 2021, the decomposition rate of the litter in the RB was significantly higher than that in the SC and CK. Five years after cutting, the decomposition rate of the litter in the three treatment plots was RB > SC > CK (*p<* 0.05).

**Table 2:** Regression analysis between dry weight residue of litter and time.

Time	Treatment	Equation	R^2^	Remaining rate/%	k	t_0.5_/a	t_0.95_/a
2017	SC	y=105.24e^-0.617t^	0.883	50.84 ± 3.81Aa	0.62 ± 0.01Aa	1.12 ± 0.02Aa	4.85 ± 0.09Aa
	RB	y=105.09e^-0.622t^	0.894	50.75 ± 2.00Aa	0.62 ± 0.00ABa	1.11 ± 0.00ABa	4.81 ± 0.02ABa
	CK	y=109.78.21e^-0.633t^	0.864	52.13 ± 1.99Aa	0.63 ± 0.04Aa	1.10 ± 0.06ABa	4.74 ± 0.26ABa
2018	SC	y=99.61e^-0.408t^	0.919	66.66 ± 7.73Ba	0.41 ± 0.08Ba	1.68 ± 0.29Ba	7.26 ± 1.25Ba
	RB	y=101.35e^-0.491t^	0.956	59.48 ± 2.73Ba	0.49 ± 0.05Ca	1.41 ± 0.14Ca	6.10 ± 0.62Ca
	CK	y=105.16e^-0.497t^	0.869	59.32 ± 6.06Aa	0.50 ± 0.08Aa	1.38 ± 0.024Aa	5.98 ± 1.04Aa
2019	SC	y=104.84e^-0.592t^	0.844	55.91 ± 8.37Aa	0.60 ± 0.12Aa	1.17 ± 0.21Aa	5.07 ± 0.92Aa
	RB	y=105.92e^-0.614t^	0.897	55.04 ± 2.99Ca	0.61 ± 0.06Ba	1.11 ± 0.10Ba	4.79 ± 0.45Ba
	CK	y=104.82e^-0.639t^	0.897	53.33 ± 5.64Aa	0.64 ± 0.15Aa	1.09 ± 0.22ABa	4.74 ± 0.95ABa
2020	SC	y=101.62e^-0.563t^	0.943	56.67 ± 1.40ABa	0.56 ± 0.01Aa	1.22 ± 0.02Aa	5.28 ± 0.10Aa
	RB	y=103.93e^-0.599t^	0.924	54.08 ± 1.63ACa	0.60 ± 0.03Ba	1.14 ± 0.06Ba	4.93 ± 0.25Ba
	CK	y=104.61e^-0.595t^	0.914	53.00 ± 2.58Aa	0.60 ± 0.04Aa	1.14 ± 0.07ABa	4.95 ± 0.31ABa
2021	SC	y=106.76e^-0.728t^	0.829	51.16 ± 5.52Aa	0.73 ± 0.09Ca	0.92 ± 0.11Aa	3.97 ± 0.47Aa
	RB	y=106.5e^-0.81t^	0.950	46.09 ± 0.31Da	0.81 ± 0.01Da	0.84 ± 0.01Da	3.62 ± 0.04Da
	CK	y=106.32e^-0.658t^	0.863	52.46 ± 5.64Aa	0.66 ± 0.14Aa	1.05 ± 0.19Ba	4.56 ± 0.85Ba

Values are the mean ± standard deviation (n=3). Different lowercase letters indicate a significant difference in litter decomposition parameters between the different treatment plots at the same time (p< 0.05); Different capital letters indicate a significant difference in litter decomposition parameters between the different times in the same treatment plot (p< 0.05).

## Discussion

### Low density reduces litter production

At the stand level, changes in nutrient return are reflected in the litter nutrient concentration and quality (Turner and Lambert, 2015), and biomass is the main factor affecting this. In this study, we found that the litter yield decreased in the SC plots the first year after cutting but gradually increased as the plots recovered. Consequently, five years after cutting, there was no significant change in the litter yield across the three treatments. As the bamboo density was significantly reduced with the SC (Zheng et al., 2022c), the litter yield was reduced in the period immediately following the cutting. Furthermore, the age structure of bamboo forests also affects litter yield. The leaves of new trees are generally changed after one year of growth, while the leaves of bamboos over one year old are changed every two years (Su, 2012). In the first year after cutting, all the bamboos in the SC were new trees, and the reduction of leaf-changing bamboos may also lead to a decrease in litter yield.

The high density of RB represents an environment of intense nutrient competition, reflected in the decrease of the number of shoots and bamboo (Zheng et al., 2022b). However, in high density, increasing the height to crown base and bamboo height is a growth strategy to obtain nutrients, which may also lead to a decrease in bamboo leaf biomass. A previous study has shown that the culm, leaf and branch productivity of RB decreased over time during strip cutting in a Moso bamboo forest (Zheng et al., 2022b). In a high-density bamboo forest, there was a decoupling relationship between productivity and soil nutrients, and high density is an important reason for the decline in productivity (Zheng et al., 2022b). Furthermore, after the formation of new bamboo, the DBH and bamboo height factors remained stable, and the growth rate of dry matter accumulation decreased significantly after the growth of II “du” (Su, 2012). The proportion of old bamboo in RB was high (Zheng et al., 2022b). After the growth of III “du,” the biomass dry mass of bamboo leaves increased little (Su, 2012). IV and V “du” bamboo are considered middle-aged bamboo; in this stage, the Moso bamboo’s physiological activities and nutrient content remain stable but will soon turn to decline (Su, 2012). Generally, VI or above “du” bamboo is considered aged; at this stage, bamboo vitality decreases, and respiratory consumption is large. Therefore, we suggest that the decrease in bamboo leaf productivity is one of the main reasons for the decrease in nutrient return in RB.

### Nutrient reabsorption efficiency affects litter nutrient content

In addition to litter yield, nutrient content is also a key factor affecting nutrient return. Previous studies have found that to achieve sufficient Moso bamboo yields nitrogen is crucial, and that the nitrogen demand and absorption are highest at each stage of growth and development (Su et al., 2019). The nitrogen in leaves can thus be transported to improve utilization efficiency before shedding to maintain the internal supply of balanced nutrients between the soil and plants (Turner and Lambert, 2013). Phosphorus is an essential element in higher plants and is usually a highly mobile and frequently translocated element (Umemura and Takenaka, 2014). Potassium accumulates in meristems and young tissues and is assimilated by the roots of higher plants (White, 2012). In addition, the leaching of rainfall was also found to have a significant influence on the concentration of potassium in bamboo leaves. Sakai and Tadaki (1997) studied the potassium concentration in the rainfall in a Moso bamboo forest and verified that potassium was leached from bamboo leaves. Therefore, we believe that the lack of significant difference in nutrient content at the early stage of harvesting is mainly due to a low nutrient reabsorption efficiency. Nutrient reabsorption refers to the transport of mobile proteins, carbohydrates, and other nutrients from aging tissues and organs to other tissues to ensure that they remain within the plant for use in physiological processes and future growth needs (Tang et al., 2013). When soil nutrient availability decreases, the internal reabsorption levels increase, which is manifested by a decrease in the nutrient concentration in the litter (Johnson and Turner, 2019). However, Zeng (2019) investigated the physical and chemical properties of bamboo forest soil after cutting with different widths, it was found that after one growing season, the soil quality of the cut plots was higher than that of the traditional management plots, indicating that strip cut improved soil fertility in the short term.

The nutrient content of the litter in the RB and SC was higher than that in the CK. On one hand, due to the high density of the bamboo and high proportion of Moso bamboo that was over 6 years old in the RB, the growth of the Moso bamboo was slow and the demand for nutrients reduced. Therefore, by reducing the transport of nutrients, more nutrients are stored in the leaves and returned to the soil. On the other hand, strip cutting may provide a sufficient nutrient for the growth of new bamboo trees by significantly decreasing stand density (Bohlen et al., 2004), thus reducing the reabsorption and utilization of nutrients (Zheng et al., 2022c).

### Nutrient cycle affects decomposition efficiency

Litter decomposition comprises a basic biogeochemical cycle in forest ecosystems (Tu et al., 2014), and the practice of obtaining SC has changed abiotic factors (Zheng et al., 2021a) and biotic factors (Zheng et al., 2022a) in Moso bamboo forests. In 2018, the decomposition rate of litters in SCs was lowest, and the annual rate of residual litter retention was significantly higher than that in other years (**Table 2**). This is related to the growth and development characteristics of a bamboo forest. According to the nutrient utilization cycle, the growth and development of Moso bamboo are divided into on- and off-years (Su, 2012). During the on-years, the female bamboo uses physiological integration to transport nutrients to the new bamboo on the whip *via* the underground rooting system, and many shoots sprout during this time (Song et al., 2016). Meanwhile, in the off-years, the germination of new bamboo shoots is reduced, and most photosynthates are used for the growth of the underground whip roots (Li et al., 2019). Moreover, most nutrients used for the growth of the bamboo root originate from photosynthesis (Zhang et al., 1999). In addition, more productivity occurred in bamboo leaves of SCs, and the photosynthetic capacity of bamboo was strong. At this point, nutrient release from litter decomposition is slower, which contributes to soil nutrient storage (Zheng et al., 2022c). However, we cannot rule out the transfer of nutrients from RBs to SCs through physiological integration (Su et al., 2019).

Five years after cutting, the litter decomposition rate of the RB and SC were the highest, and the decomposition rate occurred in the following order: RB>SC>CK (**Table 2**, *p<* 0.05). This is consistent with the order of the standing bamboo density of the three treatment plots (Zheng et al., 2022b). In a high-density bamboo forest, trees require more nutrients to maintain their growth and development (Xia et al., 2020), and there was no difference in nutrient return among the three treatment plots (**Figure 4**), which means that the RB and SC require more fertile soil (Liu, 2009). The substrate quality of the leaf litter also affected the litter decomposition rate. The experimental research results of Liu et al. (2011) on the decomposition of Moso bamboo leaf litter in Fujian found that the decomposition rate of Moso bamboo leaf litter was 0.66; however, Ren et al. (2018) found that in Sichuan, it was 0.58. Tu et al. (2014) studied litter decomposition in the bamboo ecosystem of southwest and south subtropical China and found that the initial C/N ratio significantly explained the remaining mass decomposition. In 2021, the contents of nitrogen and potassium in litters of the SC were significantly higher than those in control plots (**Figure 3**, *p<* 0.05). Previous studies have found that the nitrogen concentration in the leaf litter has a positive effect on the decomposition rate during the early stages (Tu et al., 2014). In contrast, in the later stages of decomposition, the concentration of nitrogen has a negative effect, as the N binds to lignin to form compounds that are extremely difficult to decompose (Berg and Mcclaugherty, 2013).

## Conclusion

Strip cutting reduced the bamboo density of SC, thus reducing nutrient return. With the recovery of the plot, the nutrient return level of SC reached CK. However, the nutrient return of RB did not increase after reaching a certain density owing to productivity. The decomposition rate of the litter in the SC and RB was significantly accelerated 5 years after cutting (*p<* 0.05), indicating that the nutrient turnover time was shortened. Consequently, the RB should be density managed to improve the efficiency of nutrient utilization. Nutrients should be added to the SC using fertilization methods to compensate for the nutrient loss that results from cutting and improve bamboo forest restoration.

## Data availability statement

The raw data supporting the conclusions of this article will be made available by the authors, without undue reservation.

## Author contributions

SF and FG designed this study and improved the English language and grammatical editing. YZ wrote the first draft of manuscript and performed the data analysis. YZ and XiZ did the field works. XuZ gave guidance and methodological advice. All the coauthors contributed to the discussion, revision and improvement of the manuscript.
